# Carotid Plaques and Type 2 Diabetes as Predictors of Cardiovascular Events and Mortality: Insights from the LitHiR Prospective Cohort Study

**DOI:** 10.3390/jcm15145695

**Published:** 2026-07-21

**Authors:** Vilma Dzenkeviciute, Tadas Adomavicius, Gabriele Tarutyte, Egidija Rinkuniene, Vytautas Kasiulevicius, Jolita Badariene

**Affiliations:** 1 Clinic of Internal and Family Medicine, Faculty of Medicine, Institute of Clinical Medicine, Vilnius University, LT-03101 Vilnius, Lithuania; tadas.adomavicius@mf.vu.lt (T.A.); vytautas.kasiulevicius@mf.vu.lt (V.K.); 2 Department of Research and Innovation, Faculty of Medicine, Vilnius University, 03101 Vilnius, Lithuania; gabriele.tarutyte@santa.lt; 3 Clinic of Cardiac and Vascular Diseases, Faculty of Medicine, Institute of Clinical Medicine, Vilnius University, 03101 Vilnius, Lithuania; egidija.rinkuniene@santa.lt (E.R.); jolita.badariene@santa.lt (J.B.)

**Keywords:** cardiovascular disease, carotid plaque, diabetes mellitus, all-cause mortality, ultrasound imaging, stroke, myocardial infarction, risk stratification

## Abstract

**Background/Objectives**: Type 2 diabetes mellitus (DM) and carotid atherosclerosis are established risk factors for cardiovascular disease (CVD). However, their relative and combined prognostic significance in individuals without known CVD remains uncertain. This study aimed to evaluate the independent and combined associations of carotid plaques and DM with cardiovascular events and mortality in a high-cardiometabolic-risk primary prevention cohort. **Methods**: This prospective cohort study followed 6138 participants (3571 men [57%] and 2567 women [43%]; median age 53 years, IQR 48–58) enrolled in the Lithuanian High Cardiovascular Risk (LitHiR) primary prevention program from 2006 to 2023. All individuals underwent clinical assessment and carotid ultrasonography. Participants were stratified by DM status and the presence of carotid plaques. Associations with cardiovascular events, major adverse cardiovascular events (MACE, including non-fatal ischaemic stroke, non-fatal myocardial infarction, and cardiovascular death), and all-cause mortality were evaluated using Cox proportional hazards models. **Results**: During follow-up (median 6.5 years, IQR 4.5–9.0), 954 participants (16%) experienced composite cardiovascular events (MACE, including non-fatal ischaemic stroke, non-fatal myocardial infarction, and cardiovascular death). Bilateral carotid plaques were more common in individuals with cardiovascular events (23.3% vs. 19.4%; *p* = 0.007). Individuals with both DM and carotid plaques had the highest risk of MACE (HR = 1.696; 95% CI: 1.346–2.136; *p* < 0.001), myocardial infarction (MI; HR = 4.089; 95% CI: 1.590–10.516; *p* = 0.003), and all-cause mortality (HR = 1.802; *p* = 0.015). The presence of carotid plaques without DM was independently associated with increased risk of MACE (HR = 1.234; 95% CI: 1.062–1.433; *p* = 0.006) and MI (HR = 2.492; 95% CI: 1.176–5.278; *p* = 0.017), but not all-cause mortality (HR = 0.986; 95% CI: 0.689–1.413; *p* = 0.941). In contrast, DM without carotid plaques was not significantly associated with any outcome examined. **Conclusions**: In this high-cardiometabolic-risk primary prevention cohort, carotid plaques were independently and more strongly associated with cardiovascular outcomes than diabetes alone. Individuals with both carotid plaques and diabetes showed the highest observed risk of adverse cardiovascular events and all-cause mortality. These findings are associational and apply to a high-risk metabolic syndrome cohort; extrapolation to the general population requires further study. The results support the prognostic value of carotid plaque assessment as an adjunctive tool in cardiovascular risk evaluation.

## 1. Introduction

Cardiovascular diseases (CVDs) remain the leading cause of morbidity and mortality worldwide and continue to impose a substantial public health burden despite advances in prevention and treatment. Atherosclerosis is the principal pathological process underlying myocardial infarction, ischaemic stroke, and other major cardiovascular events. Recent evidence suggests that assessment of subclinical atherosclerosis may improve cardiovascular risk stratification beyond traditional risk factors, particularly among individuals with increased cardiometabolic risk [1,2].

Contemporary concepts of atherosclerosis emphasise that plaque burden and plaque characteristics provide more clinically relevant information than carotid intima–media thickness alone. Current ultrasound recommendations support carotid plaque assessment as a useful tool for cardiovascular risk evaluation, with plaque burden demonstrating independent associations with future cardiovascular events and mortality [2,3,4].

Type 2 diabetes mellitus (T2DM) is among the most important contributors to accelerated atherosclerosis. However, cardiovascular risk among individuals with T2DM is highly heterogeneous and cannot be fully explained by diabetes status alone. Increasing evidence suggests that direct visualisation of subclinical atherosclerosis may improve identification of diabetic patients at particularly high cardiovascular risk [5,6,7].

Carotid ultrasonography is a non-invasive, widely available, and relatively inexpensive imaging modality that enables direct assessment of subclinical atherosclerosis. Recent studies have demonstrated that carotid plaque burden and total plaque area are independently associated with major adverse cardiovascular and cerebrovascular events as well as mortality, supporting their use in cardiovascular risk assessment [2,8,9,10,11].

The Lithuanian High Cardiovascular Risk (LitHiR) programme is a nationwide primary prevention initiative involving middle-aged individuals with increased cardiometabolic risk and has previously generated important data regarding cardiovascular risk factors and carotid atherosclerosis [11]. However, the comparative prognostic significance of carotid plaques and diabetes mellitus, as well as their combined associations with cardiovascular events and mortality, has not been specifically investigated.

Therefore, the aim of this study was to evaluate the independent and combined associations of carotid plaques and diabetes mellitus with cardiovascular events and all-cause mortality in a middle-aged high-cardiovascular-risk primary prevention cohort.

## 2. Materials and Methods

### 2.1. Study Design and Population

The data analysed in this research were drawn from participants enrolled in the Lithuanian High Cardiovascular Risk (LitHiR) primary prevention initiative. Launched in 2006, this government-supported programme aims to prevent the early onset of atherosclerosis among middle-aged individuals at elevated cardiovascular risk. The study focused on adults aged 40 to 65 years who had been diagnosed with metabolic syndrome and were assessed at Vilnius University Hospital Santaros Klinikos between 2006 and 2023.

Metabolic syndrome was identified when individuals met at least three of the following criteria: waist circumference of 102 cm or more for men and 88 cm or more for women; systolic blood pressure (SBP) of at least 130 mmHg and/or diastolic blood pressure (DBP) of 85 mmHg or more; fasting plasma glucose of 5.6 mmol/L or higher, or a diagnosis of type 2 diabetes mellitus; triglycerides (TG) of 1.7 mmol/L or higher; and high-density lipoprotein (HDL) cholesterol below 1.03 mmol/L for men and 1.29 mmol/L for women. Type 2 diabetes mellitus was ascertained by self-reported physician diagnosis, use of antidiabetic medications, or fasting plasma glucose ≥7.0 mmol/L at baseline examination (consistent with WHO and ADA diagnostic thresholds). Diabetes status was determined exclusively at baseline; incident diabetes developing after enrolment was not used to reclassify participants. All data were collected prospectively in line with the standardised procedures of the LitHiR programme. Variables critical to the study—such as smoking status, LDL and HDL cholesterol, total cholesterol (TC), SBP, DBP, fasting glucose, heart rate, BMI, and carotid artery ultrasound results—were extracted from the database. Only patients with complete data for each parameter were included to ensure an accurate and comprehensive cardiovascular risk assessment.

Exclusion criteria included a history of coronary artery disease, silent myocardial ischaemia, ischaemic or haemorrhagic stroke, transient ischaemic attack, peripheral arterial disease, active cancer, severe liver or renal failure, ongoing arrhythmias, significant psychiatric illness, substance abuse, and pregnancy.

#### Ethics Approval

The study was conducted in accordance with the Declaration of Helsinki and approved by the Vilnius Regional Biomedical Research Ethics Committee (Protocol No. 2019/3-1104-603, approval date 21 March 2019).

### 2.2. Data Collection

Demographic data, including age and sex, were recorded. Information on smoking habits was obtained via self-report questionnaires and classified into four categories: (a) never smoked; (b) currently smokes fewer than 10 cigarettes per day; (c) currently smokes 10 or more cigarettes per day; and (d) former smoker. Blood pressure was measured manually using a Riester precisa^®^ sphygmomanometer (Jungingen, Germany), with the cuff placed on the upper arm while the participant was seated, in accordance with the European Society of Cardiology’s recommendations for hypertension management [12]. A comprehensive evaluation of cardiovascular clinical indicators was conducted. This included measurements of heart rate, body mass index (BMI), total cholesterol (TC), high-density lipoprotein (HDL) cholesterol, low-density lipoprotein (LDL) cholesterol, and triglyceride (TG) levels. In addition, carotid ultrasound was used to measure intima–media thickness (IMT) and to detect atherosclerotic plaques. Data on any prior stroke or myocardial infarction were also recorded.

### 2.3. Carotid Plaque Assessment

Each participant in the study underwent a bilateral carotid duplex ultrasound examination. The procedure was performed with the subject supine, using the Art.Lab system (manufactured by Esaote Europe B.V., Maastricht, The Netherlands). The intima–media thickness (IMT) of the common carotid artery was measured automatically six times along a segment of at least 15 mm. Plaques were identified according to the most recent Mannheim Carotid Intima–Media Thickness and Plaque Consensus criteria [13]. According to this definition, a carotid plaque is a focal structure that protrudes into the arterial lumen by at least 0.5 mm or by 50% more than the adjacent IMT, or has an overall thickness greater than 1.5 mm. The carotid artery on both sides of the neck was segmented into four anatomical regions: the common carotid artery, the carotid bifurcation, the internal carotid artery, and the external carotid artery. Each segment was evaluated using transverse ultrasound sweeps to detect plaque formation. A score was assigned for plaque presence in each segment, and these were summed across both sides to obtain the total carotid plaque score. This score was used in the statistical analysis, with ultrasound operators blinded to participants’ cardiovascular histories to ensure objectivity. Inter-observer reliability for plaque detection was assessed in a random sample of 100 examinations, yielding a kappa coefficient of 0.89 (95% CI: 0.82–0.96), indicating excellent agreement. All examinations were performed using the same ultrasound platform (Esaote Art.Lab) throughout the study period (2006–2023), following the standardised LitHiR measurement protocol. Trained operators performed all examinations; operator training and standardisation of the measurement protocol were maintained throughout the recruitment period. Longitudinal quality-control data specific to individual time windows are not available and constitute a study limitation.

### 2.4. Follow-Up and Outcomes

Study participants were followed up through 2023, with a median follow-up of 6.5 years (interquartile range: 4.5–9.0 years). The primary endpoints were new cases of ischaemic stroke, classified under ICD-10 code I63.x, and myocardial infarction. Additionally, two secondary outcomes were evaluated: (1) a composite measure known as major adverse cardiovascular events (MACE), which included non-fatal ischaemic stroke (I63.x), non-fatal myocardial infarction (I21.x), and deaths due to cardiovascular causes (all codes beginning with I and R96); and (2) death from any cause. Outcome data were obtained from individual-level records of two official sources: the Lithuanian Patient Registry (LPR) and the Lithuanian Cause of Death Registry, both of which are compulsory reporting systems.

### 2.5. Statistical Analysis

All statistical analyses were conducted using R (version 4.2.2). Descriptive statistics summarised demographic characteristics. For continuous variables, results were presented as means with standard deviations, while categorical data were shown as counts and corresponding percentages. Comparisons of continuous variables were carried out using either Student’s *t*-test or the Mann–Whitney U test, depending on the distribution of the data. Categorical variables were analysed using Pearson’s chi-square test. The Shapiro–Wilk test was used to assess normality. For normally distributed variables, one-way analysis of variance (ANOVA) was used, whereas non-normally distributed data were examined with the Kruskal–Wallis test. The proportional hazards assumption was assessed using Schoenfeld residuals; no significant violations were detected (all *p* > 0.05).

Time-to-event analyses were performed using Cox proportional hazards regression models to evaluate the association between carotid plaque status and diabetes mellitus and the risk of cardiovascular outcomes. Models were adjusted for age, sex, smoking status, body mass index, systolic blood pressure, total cholesterol, and use of lipid-lowering and antihypertensive medications. Participants were categorised into four groups according to the presence of carotid plaques and diabetes mellitus: (1) no plaques and no diabetes (reference group), (2) plaques without diabetes, (3) diabetes without plaques, (4) both plaques and diabetes.

Hazard ratios (HRs) with 95% confidence intervals (CIs) were calculated for cardiovascular events, stroke, myocardial infarction, and all-cause mortality. All comparisons used clinically relevant reference groups, as specified for each model (e.g., individuals without carotid plaques and without diabetes for combined analyses). Complete-case analysis was used; participants with missing covariate data were excluded from multivariable models. Variance inflation factors (VIF < 5) confirmed no meaningful multicollinearity.

### 2.6. Use of Artificial Intelligence

Generative artificial intelligence tools were used for language editing and improvement of clarity. No AI tools were used in study design, data analysis, or interpretation of the results.

## 3. Results

### 3.1. Baseline Characteristics

A total of 6138 participants were included in the final analysis. The cohort comprised 3571 men (57%) and 2567 women (43%). Overall, 1101 participants (17.9%) had diabetes mellitus at baseline, corresponding to 888 participants without cardiovascular events and 213 participants with cardiovascular events during follow-up (888 + 213 = 1101). During follow-up, 954 participants (16%) experienced cardiovascular events. Participants who developed cardiovascular events were slightly older than those without events (median age 54 vs. 53 years; *p* < 0.001) and more frequently male (63.6% vs. 57.2%; *p* < 0.001). No significant differences were observed in smoking status between groups.

Small but statistically significant differences were observed in lipid parameters, including total cholesterol (6.32 vs. 6.20 mmol/L; *p* = 0.014), LDL cholesterol (4.10 vs. 4.03 mmol/L; *p* = 0.017), and HDL cholesterol (1.13 vs. 1.16 mmol/L; *p* = 0.020). Carotid intima–media thickness was higher among participants with cardiovascular events (median 671.75 vs. 659.50 μm; *p* = 0.004).

Bilateral carotid plaques were more frequent in individuals who experienced cardiovascular events (23.3% vs. 19.4%; *p* = 0.007). Other clinical variables, including blood pressure and BMI, were similar between groups. Baseline characteristics are summarised in Table 1.

### 3.2. Association of Carotid Plaques and Diabetes with Cardiovascular Events

At baseline, 1101 participants (17.9%) had type 2 diabetes mellitus and 2869 (46.7%) had carotid plaques. Participants were stratified into four groups: (a) without diabetes and without carotid plaques, (b) with diabetes but no carotid plaques, (c) with carotid plaques but no diabetes, and (d) with both diabetes and carotid plaques. These subgroups were compared with those with neither plaques nor diabetes to assess relative risks of CV events, stroke, MI, and cardiovascular mortality.

**Figure 1:**
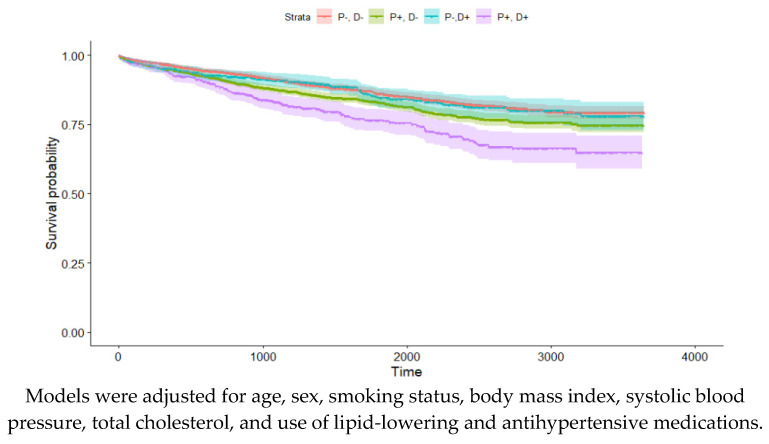
Kaplan–Meier curves for the cumulative incidence of cardiovascular events by carotid plaque and diabetes mellitus status. P−, D−: no carotid plaques and no diabetes (reference); P+, D−: carotid plaques without diabetes; P−, D+: diabetes without carotid plaques; P+, D+: both.

Cox proportional hazards regression showed that participants with both carotid plaques and diabetes had the highest risk of MACE (HR = 1.696; 95% CI: 1.346–2.136; *p* < 0.001). This group also had a significantly elevated risk of myocardial infarction (HR = 4.089; 95% CI: 1.590–10.516; *p* = 0.003) and all-cause mortality (HR = 1.802; *p* = 0.015). Individuals with carotid plaques who did not have diabetes also had a significantly higher risk of MACE (HR = 1.234; 95% CI: 1.062–1.433; *p* = 0.006) and myocardial infarction (HR = 2.492; 95% CI: 1.176–5.278; *p* = 0.017). However, no significant association with all-cause mortality was observed (HR = 0.986; 95% CI: 0.689–1.413; *p* = 0.941). Notably, this group was more than twice as likely to experience MI (HR = 2.492; 95% CI: 1.176–5.278; *p* = 0.017), although the risk of stroke did not reach statistical significance (*p* = 0.401). In contrast, diabetes alone, without plaque, was not associated with a statistically significant increase in any outcome examined. The detailed results are presented in Figure 1 and Table 2.

## 4. Discussion

In this prospective cohort of middle-aged individuals at high cardiometabolic risk without established cardiovascular disease, carotid atherosclerosis emerged as a stronger and more consistent marker of cardiovascular risk than diabetes mellitus alone. Carotid plaques were independently associated with major adverse cardiovascular events and myocardial infarction, while the combination of carotid plaques and diabetes identified the subgroup at highest risk. In contrast, diabetes without carotid plaques was not associated with increased cardiovascular risk.

These findings underscore the importance of subclinical atherosclerosis as a key determinant of cardiovascular risk. Although diabetes mellitus is traditionally regarded as a major risk factor for atherosclerotic cardiovascular disease, our results suggest that its prognostic impact is not uniform across individuals.

The present study was conducted within the LitHiR (Lithuanian High Cardiovascular Risk) primary prevention programme, which has been described in detail previously [11]. The present analysis extends our earlier publication [11]—which examined carotid plaques and hypertension as independent predictors of cardiovascular disease and all-cause mortality in the same cohort—by introducing a directly comparative framework in which carotid plaque burden and type 2 diabetes mellitus are analysed as competing and combined cardiovascular risk exposures. While the study populations overlap substantially, the research questions, primary exposures, and analytical approach differ: the present work introduces a four-group stratification according to carotid plaque status and diabetes status and, to our knowledge, represents the first analysis of this specific combination of exposures within the LitHiR cohort.

A particularly notable finding of this study is that individuals with diabetes but without carotid plaques did not have a significantly increased risk of cardiovascular events or mortality. This observation supports the view that diabetes should not be uniformly treated as a coronary artery disease equivalent, and that risk stratification within this population may benefit from additional markers of subclinical atherosclerosis [10,11]. In contrast, the presence of carotid plaques alone was associated with an increased risk of cardiovascular events and myocardial infarction, further reinforcing their prognostic relevance [14]. These findings are consistent with population-based studies demonstrating that carotid plaque score independently predicts cardiovascular events and mortality [1,3].

The combination of carotid plaques and diabetes was associated with the highest observed risk across the analysed outcomes. This finding suggests that the coexistence of metabolic abnormalities and established subclinical atherosclerosis identifies a subgroup at particularly high cardiovascular risk. However, formal interaction analyses were not performed; therefore, these findings should not be interpreted as evidence of a statistical interaction between diabetes and carotid plaques [7,13,15]. One potential explanation is that individuals with diabetes but without detectable carotid plaques may represent an earlier stage of the disease, with a shorter duration and better glycaemic control. Interestingly, carotid plaques were associated with myocardial infarction but not with stroke in the four-group analysis. This may reflect the heterogeneous aetiology of stroke, including cardioembolic, large-artery atherosclerotic, and small-vessel subtypes, which were not distinguished in the present dataset. Further studies are required to evaluate whether carotid plaque burden has differential predictive value for specific stroke subtypes.

Prior studies have specifically examined the relationship between carotid plaque burden and cardiovascular outcomes in patients with type 2 diabetes mellitus. Jeevarethinam et al. [5] demonstrated that the presence of carotid plaques independently predicted major adverse cardiovascular events in a diabetic cohort, with a markedly elevated risk of myocardial infarction and coronary revascularisation compared with plaque-free individuals with diabetes. Similarly, Cardoso et al. [6] showed in a long-term prospective analysis that carotid plaque was a strong predictor of cardiovascular outcomes in T2DM, with associations that remained significant after adjustment for traditional risk factors. Östgren et al. [7] further confirmed that the degree of carotid atherosclerosis—reflecting cumulative metabolic and vascular injury—was substantially greater in individuals with diabetes than in normoglycaemic controls, even after accounting for conventional risk factors. The present findings align with this body of evidence: participants with both carotid plaques and diabetes demonstrated the highest observed MACE hazard ratio (1.696, 95% CI 1.346–2.136, *p* < 0.001) and MI hazard ratio (4.089, 95% CI 1.590–10.516, *p* = 0.003), exceeding those observed for either exposure alone. Multiple metabolic risk factors have been shown to amplify carotid plaque formation in T2DM, including dyslipidaemia, hypertension, and fasting hyperglycaemia [16].

The higher proportion of CV events among men in our cohort is consistent with well-established sex differences in cardiovascular risk, which may reflect hormonal, behavioural, and sociocultural factors [17].

The clinical implications of these findings are substantial. Carotid ultrasound is a widely available, non-invasive, and relatively low-cost imaging modality that enables direct visualisation of subclinical atherosclerosis [5,6]. Incorporating carotid plaque assessment into systematic cardiovascular risk evaluation may improve identification of high-risk individuals, particularly among those with diabetes. The absence of carotid plaques may identify a lower-risk subgroup of individuals with diabetes and support more individualised management.

Several limitations should be acknowledged. Participants were recruited from a high-risk prevention programme, which limits generalisability to the broader population. Information on diabetes duration, HbA1c, renal function, and glucose-lowering therapy was unavailable. Carotid plaque calcification, total plaque area, and inflammatory biomarkers were not systematically assessed [18]. Residual confounding cannot be excluded because of the observational design. Finally, predictive performance metrics, including C-statistics, AUC, NRI, and IDI, were not evaluated.

## 5. Conclusions

In this prospective cohort study of middle-aged individuals at high cardiometabolic risk without established cardiovascular disease, carotid plaques were independently associated with major adverse cardiovascular events (MACE) and myocardial infarction, with stronger and more consistent associations than those observed for type 2 diabetes mellitus alone. In contrast, diabetes mellitus in the absence of carotid plaques was not significantly associated with any cardiovascular outcome examined.

The highest risk of adverse outcomes was observed among participants with both carotid plaques and diabetes mellitus. Although carotid plaques alone were associated with increased risk of major adverse cardiovascular events and myocardial infarction, they were not associated with increased all-cause mortality in the absence of diabetes.

Findings from the LitHiR high-cardiovascular-risk primary prevention cohort support carotid plaque assessment as an adjunctive tool for cardiovascular risk stratification in individuals with increased cardiometabolic risk. Because this was an observational study conducted in a selected high-risk population, the results should be interpreted as associations rather than evidence of causality. Further prospective studies incorporating diabetes duration, glycaemic control, renal function, and formal prediction-model-based analyses are warranted.

## Figures and Tables

**Table 1:** Baseline characteristics of the study population and incidence rate of adverse outcomes.

Characteristic	Population for Analysis, N = 6138		
	Without CV Event, N = 5184	with CV Event, N = 954	*p*-Value
Follow-up (years) ^‡^	6.03 (4.27, 8.94)	8.38 (5.65, 9.55)	<0.001
Age (years)	53 (48, 58)	54 (50, 59.75)	<0.001
Sex:			
Male (%)	2964 (57.18)	607 (63.63)	<0.001
Female (%)	2220 (42.82)	347 (36.37)	
Smoking:			
Non-smoker (%)	3625 (70.62)	651 (68.60)	0.5
<10 cig./day (%)	538 (10.48)	106 (11.17)	
≥10 cig./day (%)	643 (12.53)	122 (12.86)	
Quit smoking (%)	327 (6.37)	70 (7.38)	
Unknown	51	5	
Heart rate (bpm)	66 (60, 73)	65 (59, 73)	0.4
FBG (mmol/L)	6.02 (5.66, 6.55)	6.01 (5.6, 6.63)	0.6
Systolic BP (mm Hg)	138 (129, 148)	138 (129, 149)	0.8
Diastolic BP (mm Hg)	84 (77, 91)	83 (76.25, 90)	0.3
PAH:			
Yes (%)	4818 (92.94)	900 (94.34)	0.12
No (%)	366 (7.06)	54 (5.66)	
BMI	31.55 (28.93, 34.6)	31.59 (28.72, 34.67)	0.8
Diabetes: ^†^			
Yes (%)	888 (17.13)	213 (22.33)	<0.001
No (%)	4296 (82.87)	741 (77.67)	
TC (mmol/L)	6.2 (5.37, 7.12)	6.32 (5.49, 7.3)	0.014
LDL-C (mmol/L)	4.03 (3.26, 4.79)	4.1 (3.33, 4.91)	0.017
HDL-C (mmol/L)	1.16 (0.99, 1.37)	1.13 (0.97, 1.34)	0.02
CIMT	659.5 (596.5, 731)	671.75 (602.62, 742.88)	0.004
Carotid plaque:			
Yes (%)	2438 (47.03)	431 (45.18)	0.292
No (%)	2746 (52.97)	523 (54.82)	
Carotid plaques on both sides:			
Yes (%)	1008 (19.44)	222 (23.27)	0.007
No (%)	4176 (80.56)	732 (76.73)	
MI:			
Yes (%)	0 (0)	49 (5.14)	<0.001
No (%)	5184 (100)	905 (94.86)	
Stroke:			
Yes (%)	0 (0)	128 (13.42)	<0.001
No (%)	5184 (100)	826 (86.58)	
CV death:			
Yes (%)	24 (0.46)	18 (1.89)	<0.001
No (%)	5160 (99.54)	936 (98.11)	

Abbreviations: BMI, body mass index; BP, blood pressure; CIMT, carotid intima–media thickness; CV, cardiovascular; FBG, fasting blood glucose; HDL-C, high-density lipoprotein cholesterol; LDL-C, low-density lipoprotein cholesterol; MI, myocardial infarction; PAH, primary arterial hypertension; TC, total cholesterol. Data are presented as median (IQR) for continuous variables and n (%) for categorical variables. ^†^ Diabetes: Without CV event n = 888 (17.1%); with CV event n = 213 (22.3%); total n = 1101 (17.9%). ^‡^ Follow-up: 6.03 yr (IQR 4.27–8.94) without CV events; 8.38 yr (IQR 5.65–9.55) with CV events; overall median 6.5 yr (IQR 4.5–9.0).

**Table 2:** Multivariable Cox regression analysis for adverse outcomes.

Characteristic	MACE HR (95% CI)	*p*-Value	Stroke HR (95% CI)	*p*-Value	MI HR (95% CI)	*p*-Value	All-Cause Mortality HR (95% CI)	*p*-Value
Plaques (yes), no diabetes	1.234 (1.062; 1.433)	0.006	1.191 (0.792–1.790)	0.401	2.492 (1.176; 5.278)	0.017	0.986 (0.689–1.413)	0.941
No plaques, with diabetes	1.070 (0.824; 1.390)	0.611	1.105 (0.557–2.193)	0.776	0.378 (0.046; 3.129)	0.367	1.265	0.381
Plaques (yes), with diabetes	1.696 (1.346; 2.136)	<0.001	1.286 (0.669–2.472)	0.450	4.089 (1.590; 10.516)	0.003	1.802	0.015
Plaques (yes), (regardless of diabetes)	1.300 (1.137; 1.486)	<0.001	1.185 (0.824; 1.704)	0.360	3.287 (1.647; 6.561)	<0.001	1.102 (0.815; 1.491)	0.528
Diabetes (regardless of plaques)	1.231 (1.027; 1.476)	0.025	1.092 (0.661; 1.801)	0.732	1.402 (0.658; 2.985)	0.381	1.551 (1.067; 2.256)	0.021

Abbreviations: CV, cardiovascular; HR, hazard ratio; MI, myocardial infarction; MACE, major adverse cardiovascular events (composite of non-fatal ischaemic stroke [ICD-10: I63.x], non-fatal myocardial infarction [I21.x], and cardiovascular death [codes beginning with I and R96]). Reference group for all models: no carotid plaques and no diabetes. All-cause mortality (final column) is death from any cause and is distinct from cardiovascular mortality included within MACE. Stroke CIs for rows 1–3 reflect the precision of estimates with limited event counts per subgroup and should be interpreted cautiously. Confidence intervals were unavailable for all-cause mortality estimates in the ‘No plaques, with diabetes’ and ‘Plaques (yes), with diabetes’ groups because only summary model outputs were retained.

## Data Availability

The data presented in this study are not publicly available due to privacy and ethical restrictions. The data may be available from the corresponding author upon reasonable request.

## Abbreviations

The following abbreviations are used in this manuscript:

BMI	Body mass index
CI	Confidence interval
CIMT	Carotid intima–media thickness
CV	Cardiovascular
CVD	Cardiovascular disease
DBP	Diastolic blood pressure
HDL-C	High-density lipoprotein cholesterol
HR	Hazard ratio
IMT	Intima–media thickness
LDL-C	Low-density lipoprotein cholesterol
MACE	Major adverse cardiovascular events
MI	Myocardial infarction
SBP	Systolic blood pressure
TC	Total cholesterol
TG	Triglycerides
T2DM	Type 2 diabetes mellitus

## References

[1] Brunner G., Virani S.S., Sun W., Liu L., Dodge R.C., Nambi V., Coresh J., Mosley T.H., Sharrett A.R., Boerwinkle E. (2021). Associations Between Carotid Artery Plaque Burden, Plaque Characteristics, and Cardiovascular Events: The ARIC Carotid Magnetic Resonance Imaging Study. JAMA Cardiol..

[2] Johri A.M., Nambi V., Naqvi T.Z., Feinstein S.B., Kim E.S.H., Park M.M., Becher H., Sillesen H. (2020). Recommendations for the Assessment of Carotid Arterial Plaque by Ultrasound for the Characterization of Atherosclerosis and Evaluation of Cardiovascular Risk: From the American Society of Echocardiography. J. Am. Soc. Echocardiogr..

[3] Ihle-Hansen H., Vigen T., Berge T., Walle-Hansen M.M., Hagberg G., Ihle-Hansen H., Thommessen B., Ariansen I., Røsjø H., Rønning O.M. (2023). Carotid plaque score for stroke and cardiovascular risk prediction in a middle-aged cohort from the general population. J. Am. Heart Assoc..

[4] Lorenz M.W., Polak J.F., Kavousi M., Mathiesen E.B., Völzke H., Tuomainen T.-P., Sander D., Plichart M., Catapano A.L., Robertson C.M. (2012). Carotid intima-media thickness progression to predict cardiovascular events in the general population (the PROG-IMT collaborative project): A meta-analysis of individual participant data. Lancet.

[5] Jeevarethinam A., Venuraju S., Dumo A., Ruano S., Rosenthal M., Nair D., Cohen M., Darko D., Lahiri A., Rakhit R. (2018). Usefulness of Carotid Plaques as Predictors of Obstructive Coronary Artery Disease and Cardiovascular Events in Asymptomatic Individuals with Diabetes Mellitus. Am. J. Cardiol..

[6] Cardoso C.R.L., Salles G.C., Leite N.C., Salles G.F. (2019). Prognostic impact of carotid intima-media thickness and carotid plaques on the development of micro- and macrovascular complications in individuals with type 2 diabetes: The Rio de Janeiro type 2 diabetes cohort study. Cardiovasc. Diabetol..

[7] Östgren C.J., Otten J., Festin K., Angerås O., Bergström G., Cederlund K., Engström G., Eriksson M.J., Eriksson M., Fall T. (2023). Prevalence of atherosclerosis in individuals with prediabetes and diabetes compared to normoglycaemic individuals-a Swedish population-based study. Cardiovasc. Diabetol..

[8] Genkel V., Kuznetsova A., Lebedev E., Salashenko A., Savochkina A., Nikushkina K., Pykhova L., Sumerkina V., Shaposhnik I. (2023). Carotid total plaque area as an independent predictor of short-term subclinical polyvascular atherosclerosis progression and major adverse cardiac and cerebrovascular events. Ther. Adv. Cardiovasc. Dis..

[9] Liu Y., Kong Y., Yan Y., Hui P. (2024). Explore the value of carotid ultrasound radiomics nomogram in predicting ischemic stroke risk in patients with type 2 diabetes mellitus. Front. Endocrinol..

[10] Fabregat-Andrés O., Pérez-de-Lucía P., Vallejo-García V.E., Vera-Ivars P., Valverde-Navarro A.A., Tormos J.M. (2024). New atherogenic index for the prediction of carotid atherosclerosis based on the non-ultrasensitive C-reactive protein/HDL ratio. Clin. Investig. Arterioscler..

[11] Dženkevičiūtė V., Adomavičius T., Tarutytė G., Rinkūnienė E., Kasiulevičius V., Badarienė J. (2024). Carotid Plaques and Hypertension as Risk Factors for Cardiovascular Disease and All-Cause Mortality in Middle-Aged Adults. J. Clin. Med..

[12] Williams B., Mancia G., Spiering W., Agabiti Rosei E., Azizi M., Burnier M., Clement D.L., Coca A., de Simone G., Dominiczak A. (2018). 2018 ESC/ESH Guidelines for the management of arterial hypertension: The Task Force for the management of arterial hypertension of the European Society of Cardiology (ESC) and the European Society of Hypertension (ESH). Eur. Heart J..

[13] Touboul P.J., Hennerici M., Meairs S., Adams H., Amarenco P., Bornstein N., Csiba L., Desvarieux M., Ebrahim S., Fatar M. (2007). Mannheim Carotid Intima-Media Thickness Consensus (2004–2006): An Update on Behalf of the Advisory Board of the 3rd and 4th Watching the Risk Symposium 13th and 15th European Stroke Conferences, Mannheim, Germany, 2004, and Brussels, Belgium, 2006. Cerebrovasc. Dis..

[14] Inaba Y., Chen J.A., Bergmann S.R. (2012). Carotid plaque, compared with carotid intima-media thickness, more accurately predicts coronary artery disease events: A meta-analysis. Atherosclerosis.

[15] Liu Z., Zhang L., Sun B., Ding Y. (2024). Association of cardiovascular risk factors and intraplaque neovascularization in symptomatic carotid plaque. Front. Neurol..

[16] Yang W.L. (2023). Comprehensive analysis of risk factors for carotid plaque in type 2 diabetes mellitus: A meta-analysis. Glob. J. Med. Health Sci..

[17] Feraldi A., Zarulli V., Buse K., Hawkes S., Chang A.Y. (2025). Sex-disaggregated data along the gendered health pathways: A review and analysis of global data on hypertension, diabetes, HIV, and AIDS. PLoS Med..

[18] Noflatscher M., Schreinlechner M., Sommer P., Kerschbaum J., Berggren K., Theurl M., Kirchmair R., Marschang P. (2018). Influence of Traditional Cardiovascular Risk Factors on Carotid and Femoral Atherosclerotic Plaque Volume as Measured by Three-Dimensional Ultrasound. J. Clin. Med..

